# Perspectives of substitute decision‐makers and staff about person‐centred physical activity in long‐term care

**DOI:** 10.1111/hex.13381

**Published:** 2021-11-08

**Authors:** Charlene H. Chu, Amanda M. L. Quan, Freya Gandhi, Katherine S. McGilton

**Affiliations:** ^1^ Lawrence S. Bloomberg Faculty of Nursing University of Toronto Toronto Ontario Canada; ^2^ Institute for Life Course and Aging University of Toronto Toronto Ontario Canada; ^3^ KITE‐Toronto Rehabilitation Institute University Health Network Toronto Toronto Ontario Canada; ^4^ Dalla Lana School of Public Health University of Toronto Toronto Ontario Canada

**Keywords:** care processes, long‐term care, nursing homes, person‐centred care, physical activity, resident‐centred care

## Abstract

**Introduction:**

This paper aims to explore the care processes that best exemplify person‐centred care during physical activity (PA) for long‐term care (LTC) residents with dementia from the perspectives of substitute decision‐makers (SDMs) and LTC home staff. Little is known about how person‐centred care is reflected during PA for residents with dementia, or the barriers and benefits to enacting person‐centred care during PA.

**Methods:**

Semistructured interviews were used to collect SDMs and LTC home staffs' perspectives on the importance of person‐centred care during PA from two LTC homes in Canada. The McCormack and McCance person‐centredness framework was used to guide thematic content analysis of responses.

**Results:**

SDM (*n* = 26) and staff (*n* = 21) identified actions categorized under the *sympathetic presence* or *engagement* care processes from the person‐centredness framework as most reflecting person‐centred care. Benefits of person‐centred care during PA were categorized into three themes: functional and physical, behavioural and communication and psychosocial improvements. Barriers to person‐centred care during PA identified were lack of time, opportunities for meaningful activity in LTC setting and staff experiences with resident aggression.

**Significance:**

Understanding the care processes that are most recognized as person‐centred care and valued by SDMs and LTC home staff has implications for education and training. Insights into SDMs' care expectations regarding person‐centred care can inform staff about which actions should be prioritized to meet care expectations and can foster relationships to the benefit of residents with dementia.

**Patient and Public Contribution:**

Study participants were not involved in the development of research questions, research design or outcome measures of this study.

## 1 INTRODUCTION

Person‐centred care is a foundational principle in health policy and practice, especially within long‐term care (LTC) homes (also known as nursing homes, residential aged care facilities or care homes).^1^ Person‐centred care approaches are based on residents' needs, preferences, values and life history, and thus should guide interactions between healthcare professionals and residents to improve healthcare quality and safety.^1^, ^2^ Person‐centred care is especially pertinent to the care of residents with dementia in LTC homes as it means applying individual preferences to ‘guide all aspects of their care, supporting their realistic health and life goals’.^3^ Person‐centred care approaches significantly improve quality of life, neuropsychiatric symptoms and agitation among people with dementia.^4^ Evidence suggests that not espousing person‐centred care may increase responsive behaviours in LTC homes,^5^ in particular, during times of increased physical exertion that may induce stress.^6^

Substitute decision‐makers (SDMs)^7^ are family or friends that are often involved in care decisions, advocate for residents and communicate care expectations to LTC home staff. LTC home staff serve as the primary caregivers for residents and are very familiar with residents as they are responsible for helping to meet their daily care needs, including physical activity (PA). One resident will be cared for by several staff members throughout a 24‐h period, and each staff member often cares for eight or more residents per shift. SDMs are firsthand witnesses of whether staff espouse the values of person‐centred care with their loved ones; however, there is a paucity of empirical studies describing person‐centred care from the perspective of these stakeholders despite their critical role as advocates for their loved ones living in LTC. There is scant literature on the perceptions of SDMs on person‐centred care as a general concept, not related to care tasks.^8^ The authors reported that SDMs described person‐centred care as ‘contributing to promoting a continuation of self and normality’ (p. 4) that could be accomplished through staff providing meaningful activities and welcoming family.^8^ Currently, the ways in which person‐centred care approaches can be espoused with residents with dementia during PA are unclear.

### 1.1 Importance of PA for residents with dementia

PA is essential in LTC homes' programming,^9^, ^10^ and is crucial in preventing functional decline in residents.^11^ The benefits of PA in residents with dementia include improved strength, coordination, cognitive functioning^12^, ^13^ and extensive mobility benefits such as increased step counts.^14^ Further, PA provides cognitive and social stimulation to older adults with dementia and reduces depressive symptoms,^15^ responsive behaviours,^16^ agitation,^17^ boredom, restlessness and apathy.^18^ PA is referred to as the ‘ultimate medicine’ for older adults including those living in LTC homes.^9^

Despite the benefits associated with PA,^12^, ^16^ responsive behaviours of dementia, including agitation, reduced emotional affect and motivation and depression, can result in reluctance to engage in PA. Evidence demonstrates that older adults with dementia are significantly less physically active (e.g., less steps taken per day),^16^ show more gait impairments (e.g., irregular cadence)^19^ and consequently, lose functional mobility sooner than residents without dementia.^20^ Clinical care staff who are responsible for engaging residents with dementia in standardized PA routines report these responsive behaviours as practical challenges that inhibit residents' participation.^21^, ^22^, ^23^

Walking PA programmes for residents with dementia can be optimized to meet cognitive and physical abilities while incorporating resident goals.^24^ Previous research that examined individualized PA interventions for those with dementia were found to be moderately effective in maintaining cognitive function and reducing depression and risk of falls and disability, in addition to increasing cardiovascular function and improving well‐being.^25^, ^26^, ^27^ However, within the body of literature about PA for residents with dementia, specificities are lacking on how LTC home staff can individualize PA for this high‐risk group. Strategies for individualization are critical to the reproducibility of interventions.^28^ Further understanding of individualized PA interventions will increase the uptake of research findings into daily practice.^29^, ^30^ This information would help establish the degree to which staff's care behaviours align with person‐centred care.^28^

There has been a paucity of research examining how the person‐centred care approach can be applied to individualize PA for residents with dementia. Current literature about person‐centred care related to PA identifies it as being ‘non‐specific and varied’ (p. 109) in its implementation and its operationalization.^31^ Systematic reviews about person‐centred care as a concept reveal a lack of consistency around how it is applied in practice, contributing to the limited advice available to healthcare professionals (e.g., physiotherapists, nursing) and LTC home staff (e.g., recreational therapists, activation staff) who provide direct care.^4^, ^31^, ^32^, ^33^ Existing literature highlights that the focus and emphases placed on the themes within person‐centred care differ across types of healthcare providers and may thus hinder successful implementation of patient‐centred care in practice.^34^ A plethora of seminal texts such as policy documents, medical and nursing studies and concept analyses on the subject of person‐centred care exist,^35^, ^36^, ^37^, ^38^ but do not maintain the primacy of the patient perspective in person‐centred care. Explicit examination of person‐centred care approaches with residents and/or those closest to them, their SMDs and care staff, is needed.

### 1.2 Theoretical framework

**Figure 1:**
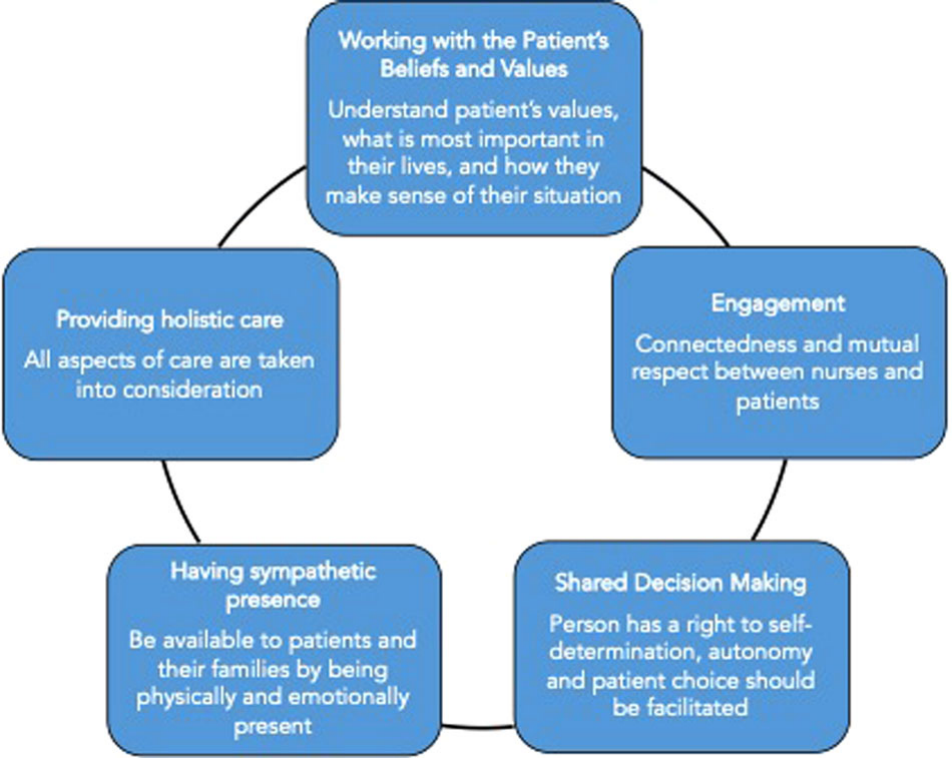
Care processes of McCormack and McCance person‐centred care framework

McCormack and McCance's person‐centredness framework is a widely used framework in nursing and healthcare that situates the development and practice of person‐centredness in a variety of healthcare contexts.^39^, ^40^ The framework describes the factors required to deliver person‐centred care (e.g., skills), the care environment that is supportive of staff and the care processes that outline various activities to deliver person‐centred care and then links these constructs to outcomes (e.g., satisfaction with care) (see Figure 1). Specifically, the core of this framework is made up of care processes: engagement, having a sympathetic presence, shared decision‐making and valuing the beliefs and values of residents. The actions and strategies aligned with, and in the spirit of, these care processes contributes to person‐centredness. This study is focused on the care processes related to PA.

Understanding which care processes during PA for residents with dementia is important, given the significant role of PA in maintaining health for older adults living in LTC homes. The actions and strategies that can be used to deliver person‐centred care in PA need to be highlighted to support the incorporation of person‐centred care into PA delivered in LTC settings.^31^ To this end, the perspectives of SMDs and staff can provide critical knowledge on how to operationalize person‐centred care when providing PA to residents with dementia. In addition, a greater understanding about SDMs' care expectations can prevent misalignment of care expectations between SDMs and staff, and discrepancies can be targeted for quality improvement. A comprehensive understanding of the behaviours that SDMs view as most meaningful and representative of person‐centred care will support LTC home staff in providing better care overall. Shared health and care expectations are critical to fostering ‘good relationships’ between LTC home staff and family members, and are critical in the well‐being of the resident.^41^ Different opinions on care expectations, specifically what care is appropriate for residents (e.g., how PA should be delivered), are a major source of conflict between staff and family members.^42^, ^43^ The implications of poor staff and SDMs relationship can increase staff burnout and depression in family caregivers.^44^, ^45^ The aim of this study is to gain insights into the SDMs and LTC home staff's perspectives about which aspects of care processes best reflect person‐centred care when delivering PA programmes. The factors required to deliver person‐centred care as identified by McCormack and McCance's framework will be used to guide our analyses. The secondary aims are to understand the perceived barriers to and benefits of implementing person‐centred care in PA.

### 1.3 Study design

This qualitative study was part of a larger mixed‐methods study of a PA intervention for LTC home residents with dementia.^46^ The PA intervention consisted of individualized regimes and behavioural and communication care plans that were developed after resident observation and in‐depth interviews with SDMs about the personhood of the resident. The results of the Multifaceted Walking Intervention, including mobility and functional outcomes, are published elsewhere.^24^ The aim of the current study was to gain insights from the perspectives of SDMs and LTC home staff on the importance of person‐centred care during PA in addition to the identification of aspects of the PA that were considered person‐centred.

The study took place in two nonprofit LTC homes in Ontario, Canada. Home 1 is a large 350‐bed facility, and Home 2 is a mid‐sized facility with 128 beds. PA throughout the study was standardized across the homes—during the 2‐month control phase, usual care and programming met the requirements of Ontario's Long‐Term Care Homes Act, 2007,^47^ and PA during the intervention phase followed the methodology published elsewhere.^24^ Staff were eligible to participate in this study if they were the primary LTC home staff (i.e., personal support worker [PSW] assigned to residents enrolled in the intervention). Note that while the care team in LTCs consists of a number of healthcare professionals, PSWs represent up to 90% of the direct care workforce in LTC.^48^ This is the case in Canada as well, where PSWs are the largest proportion of employees in LTC^49^; they are primarily responsible for the care of residents, and thus were largely targeted for study participation.

SDMs were eligible to be part of the study based on the inclusion of their loved one in the larger mixed‐methods study, which required residents to be newly admitted to the home (i.e., less than 3 months), diagnosed with dementia and able to walk for at least 2 min with or without a gait aid.^46^ SDMs and LTC home staff were approached by the PI (C. H. C.) to participate in the study after resident screening, and their associated resident was enrolled into the study. There were no specific inclusion or exclusion criteria for SDMs or staff in this study. The overall recruitment rate of SDMs and LTC home staff was 100%. The PI, a Registered Nurse with specialist training in gerontology and dementia care, was responsible for the development of the intervention and conducting the walking sessions. All participants were provided with the consent form and an in‐depth explanation about the purpose of the study and their participation. Participants were provided with ample opportunity to ask the PI questions. The PI ensured that SDMs and LTC home staff understood that their participation was voluntary. The participants could ask questions or withdraw at any point in the study without affecting their employment or care for the resident. University of Toronto Research Ethics Board approved the study (REB Protocol 14‐7737‐DE), and all participants in this study provided informed consent.

## 2 METHODS

### 2.1 Data collection

The semistructured interviews were focused on person‐centred care, the aspects that reflected person‐centred care, the challenges of providing person‐centred care for residents with dementia and the perceptions on why it is important to dementia care.^50^ Interview questions (Supporting Information Appendix A) were developed to be open‐ended, inquiring about person‐centred care processes, and included accompanying probes to elicit more detailed responses. Questions were pilot‐tested with an LTC home staff member before interviews to ensure clarity of the questions. Interviews represented an appropriate methodology for collecting the views of LTC home staff and SDMs about the acceptability of the Multifaceted Walking Intervention, the importance of person‐centred care and what actions were considered person‐centred to them.^51^ Additionally, a questionnaire was used to collect SDMs' demographic information (e.g., age, sex, years of education) and descriptive information including occupation, number of visits per week and how well they knew the resident, self‐reported, on a scale of 1–10 (10 was ‘no one knows this person more than me’) (see Supporting Information Appendix B). Similarly, LTC home staff were asked to provide their age, sex, years of education, position in the home and number of years they worked in the home.

Interviews were audio‐recorded, depending on the availability of the participant (e.g., accommodating staff breaks), and were conducted in a quiet room in the nursing home. Interviews were approximately 30 min long to accommodate staff break times and to be considerate to the SDMs who were primarily in the LTC home to visit their loved ones. The semistructured interview was conducted at the end of the Multifaceted Walking Intervention (4 months in duration) to gather in‐depth data about their perspectives by the PI, who is a doctoral prepared Registered Nurse, with formal training in qualitative interview methods and expertise in dementia care as part of her PhD. Data collection continued until data saturation was reached and no new concepts or information emerged.^52^

### 2.2 Data analysis

All interviews were transcribed verbatim, and the PI checked against the original recording for accuracy. Open‐ended questions and answers were entered into a word document. The data were analysed using a modified thematic analysis approach.^53^, ^54^ This approach involved three steps: data familiarization through close data reading, data codification and classification according to areas of exploration and theme identification through data source triangulation where similar codes are collapsed to formulate themes that emerge from the coding. This deductive approach was deemed appropriate considering the objective of the study and the predetermination to use the framework by McCormack and McCance.^55^ In the early phase of the analysis, the researchers realized the similarities between the SDMs and staff interviews and the decision was made to combine the interviews and code both groups together.

McCormack and McCance's person‐centredness framework was identified a priori to use for the analysis.^39^ An iterative process was used to code all the transcripts to generate an understanding about the activities related to each person‐centred care process in the McCormack and McCance's person‐centredness framework. Staff and SDMs referenced the Multifaceted Walking Intervention as well as general PA offered in the LTC home to residents with dementia. Two members (C. H. C., K. M.) of the research team read the interview transcripts and each independently coded the transcripts for care activities and behaviours that reflected person‐centred care during PA, and then the members discussed the codes, and categorized them corresponding to the care processes in the McCormack and McCance framework. The codes were then grouped to develop themes under each care process from the framework. Any discrepancies in interpretation were resolved through discussion. QDA Minor was used for electronic coding. To ensure methodological rigour and trustworthiness of the analysis, the following strategies were used: multiple team members participated in data analysis, data source triangulation that led to a more comprehensive understanding of the benefits and barriers related to providing person‐centred care^55^ and a dependability audit,^56^ consisting of audio recordings, transcripts, interview guide and data analysis processes and products, all documented in detail to allow outsider assessment of theoretical generalizability.

## 3 RESULTS

The characteristics of the staff and SDMs are summarized in Table 1. The majority of SDMs were daughters of residents, had a mean age 59 years and were university educated. The LTC home staff were PSWs who were predominantly female, and had been employed at the home for an average of 9 years.

**Table 1:** Participant demographics (*N* = 47)

	Substitute decision‐makers (*N* = 26)		LTC home staff (*N* = 21)	
Characteristic	*N*	%	*N*	%
Age^a^	59 (12.3), 41–86	‐	46.1 (10.7), 23–65	‐
Gender				
Male	5	19.2	2	9.6
Female	21	80.8	19	90.4
Ethnicity				
Caucasian	25	96.2	‐	‐
Other	1	3.8	‐	‐
Education level				
Grade school	1	3.8	‐	‐
High school graduate	2	7.7	‐	‐
College graduate^b^	4	15.4	20	95.2
University graduate^b^	13	50	1	4.7
Graduate school (Master's or Doctorate)	6	23	‐	‐
Employment^a^				
Number of years in healthcare	‐	‐	14 (7.2), 1–30	‐
Years of employment at facility	‐	‐	9 (3.7), 0.25–13	‐
Job status^a^				
Full‐time	‐	‐	18 (85.7)	‐
Part‐time	‐	‐	3 (14.3)	‐
Private full‐time caregiver^c^	‐	‐	5 (23.8)	‐
Average visits per week^a^	2.3 (1.7), 0.25–5	‐	‐	‐
‘How well do you know the resident?’^a^, ^d^	9.3 (1.3), 4–10	‐	‐	‐

Abbreviation: LTC, long‐term care.

^a^ Mean (SD), and range provided in lieu of *N* (%).

^b^ For LTC Home staff, college education refers specifically to completion of a healthcare aide certificate programme or RPN programme. University graduate refers to a baccalaureate degree in nursing.

^c^ Hired full‐time caregivers who were paid by the family to provide care for only their loved one.

^d^ On a scale of 1–10.

Quotations from the participants are presented in this section, and the notation indicates whether they are staff or an SDM for a resident who was assigned a pseudonym (e.g., R3).

### 3.1 Care processes: Having a sympathetic presence and engagement

From the interviews with SDMs and LTC home staff about person‐centred care, activities and behaviours from two domains from McCormack and McCance's person‐centred nursing practice framework^39^ were identified as relevant when providing PA to residents with dementia. The domains were having a sympathetic presence, defined as ‘recognizing the uniqueness and value of the individual by appropriately responding to cues through providing reassurance and showing concern’,^39^ and engagement, determined by gathering knowledge of the person and the connectedness of the practitioner with the resident.^39^ The three other care processes of the McCormack and McCance person‐centred care framework (Figure 1) were not evident among participant responses. After the analysis, the three relevant actions that emerged that were most reflective of the ‘sympathetic presence’ care process were ‘Recognizing and acknowledging resident's remaining abilities’, ‘Tailoring care to residents' preferences and capabilities’ and ‘Enabling resident's need to be independent’. These actions and examples of supporting quotes for each care process are presented in Table 2. The second care process that emerged as relevant with PA was engagement, which meant that the care provider was committed to individualizing all aspects of the PA. The two activities related to engagement were ‘Connecting with the resident’ and ‘Undivided one‐on‐one care and attention’. SDMs recognized the processes used to gather information about aspects of the residents' life histories before the PA as well as the effort to make sure that residents were asked what they wanted to do during the PA. SDMs and LTC home staff both felt that the one‐on‐one format of PA was beneficial compared to the traditional group exercise activities offered. The undivided attention between care provider and resident enabled meaningful personal connection and they noticed that residents felt more confident and uplifted after one‐on‐one PA.

**Table 2:** Quotations illustrating domains of the McCormack and McCance's person‐centred care nursing practice framework

Care processes of person‐centred care
Having sympathetic presence
*Recognizing and acknowledging residents' remaining abilities*
‘[Person‐centered care] Provided my mother with one‐on‐one attention… Living in the moment is how she sees the world right now, and the moments [the PA] provided her were very special… Consideration to when and where my mom wanted to go on a walk was taken into account and her stories and past histories were used to remind her of the neighborhood where she used to live.’ (SDM, Interviewee R4)
‘My mom is a “wanderer” and I encourage her to move around. She just doesn't know how to articulate where she is going. The researcher's approach was tailored and assumed she had remaining abilities’. (SDM, Interviewee R3)
*Tailoring care to residents' preferences and capabilities*
‘The communication [during the PA] was centered on the resident. The [PA sessions] took into consideration her capabilities, preferences, for example, staying inside or going outside, walking on the pavement surface versus the garden with grass, sunlight or shade…. It recognized the preferences and uniqueness of each resident and focused on those aspects to get them up and walking.’ (SDM, Interviewee R27)
‘Walking [as a PA] is a way to physicalize the frustration and get out of her head and rejoin the world around her. She is stuck in here on the unit within this residence. She wants to be around people and talk to them. Walking and talking about things she knows and likes is necessary in the quality of life of residents who have dementia. This is a good thing and it increases their social engagement’. (SDM, Interviewee R3)
*Enable residents' need to be independent*
‘The more she walks, the stronger she will be, so she can be more independent. She needs some help now and the activities here only provide seated exercise, so the person‐centered walking will help her maintain her standing and walking abilities more, for a longer period of time; it will keep her from not drifting away so she's forced to use her mind and use her legs. It encourages her independence during and after the physical activity’. (SDM, Interviewee R10)
Engagement
*Connecting with the resident*
‘[personal connection] is very important because if there is someone with her she feels more secure to assist her and accompany her. She feels much more confident. Her safety and protection from falls is very important’. (LTC home staff, Interviewee 21)
‘I think this [PA] was person‐centered because I was asked about how I interact with [the resident]. The intervention was one‐on‐one and so you were able to listen and ask her questions about her life. Because you got to know her and her family, she felt comfortable with you’. (SDM, Interviewee R28)
*Undivided one‐on‐one care and attention*
‘My mother enjoys walking and complains of loneliness. Combining person‐centered care and PA is an ideal intervention and all staff need to be able to do this if they are working with residents who have dementia. The one‐on‐one aspect keeps individuals more engaged, physically and mentally. I like that you moved outside… because my mother loves being outdoors’. (SDM, Interviewee 10)
‘The [one‐on‐one] attention it gives her helps support a better prognosis of the disease and gives her a sense of security. The [resident] is less destructive and can focus on her needs and wants’. (LTC home staff, Interviewee R1)
‘Consideration to when and where my mom wanted to go was integrated into the PA. My mom enjoyed talking about each of her pictures with the interventionist and being reminded of who everyone is. All the information I provided was used to engage with my mom. It was personalized perfectly to her’. (SDM, Interviewee 4)

### 3.2 The perceived benefits of person‐centred care in PA for residents with dementia

After implementing person‐centred care in PA for older adults living with dementia in the LTC homes, all the SDMs and staff reported witnessing positive resident changes. Noticeable improvements were categorized into three themes: functional (e.g., toileting, transferring, eating), behavioural (e.g., agitation), communication and psychosocial (e.g., speech, mood). Functional improvements were noted by SDMs and LTC home staff, who recognized that after the PA, residents were more involved in their own activities of daily living. This increase in function was noted as a clear benefit from the PA:

I noticed they could walk to the washroom instead of sitting and waiting like they would usually, and they could do more things during their day. They'd have more choices. (LTC home staff, interviewee R14)

LTC home staff found that residents had more confidence and improved posture to do activities of daily living, such as toileting, after engaging in PA that was delivered with person‐centred care. One staff member noticed minor improvements during and after increased PA:

More PA [helped] in toileting because she has more confidence in standing from sitting (getting up off her chair). She's less wobbly, she's more stabilized in her posture. (LTC home staff, interviewee R29)

LTC home staff and SDMs reported that residents were more willing to engage in the activities offered throughout the rest of the day. For example, the SDMs and staff for the same resident noticed an improvement in the resident's eating habits and commented:

Her eating has improved, the mobility helps her increase her metabolism and so she eats more. She needs more energy mentally and physically, so now she can eat. (SDM, interview R26)

Yes, it really maintained her mobility…With this program, she's improved her eating, she's eating bigger portions, more now than before. Before she would look at the plate and not touch the food, sometimes take the food outside into the hallway, but now she's hungry and she will eat it. (LTC home staff, interview R26)

Second, LTC home staff noticed less responsive behaviours, wherein residents were less agitated after the PA sessions:

She used to get really agitated in the dining room and yell at other people but now that had subsided with more walking. She's easier to talk to, before she would fight with people and violent attacks. The person‐centered walking gave her an outlet and was planned according to her behaviors and focused on her needs and likes. (LTC home staff, interviewee R3).

Both SDMs and staff indicated that the PA provided a protected time for social engagement, and when coupled with residents' preferences, this may have mitigated responsive behaviours. SDMs believed that deeper human connection helped to provide social engagement that was otherwise missing. One SDM spoke of their father:

My father's history, his other behaviors, his paranoia and these things were taken into account by the interventionist. Also, the time of day to most benefit my father was arranged for the one‐on‐one walks and gave him a chance to express himself, the best he could—that were remarkable. I only saw positive benefits. It's helped give him one‐on‐one attention—which he craved, and this helped reduce negative behaviors later on in the day. (SDM, R13).

Communication and psychosocial domains also improved. SDMs and staff described that because the PA was adapted to the residents' communication abilities, residents were encouraged to participate and communicate, giving them a sense of self‐confidence. Most SDMs of the residents noticed improved moods and increased self‐esteem:

The [resident felt] more useful, self‐assured and confident that they can participate in their personal path. (SDM, interviewee R29)

She can walk, but the engagement can help distract and break the day for her so she's just not walking around the nursing station. She can't verbalize anymore, and she doesn't realize what she's doing any more. Having interaction and talking to another human being will help because she wouldn't get any interaction otherwise here. (SDM, R6)

Additionally, the creation of opportunities for meaningful social engagement between the resident and the interventionist supported positive social behaviours throughout the day:

[The PA sessions are] promoting my mom's memory so she doesn't forget to put on the breaks of her walker…The [PA sessions] meant there were consistent visits from the same non‐family person. Her social skills also improved, and boredom and her depression decreased. Over the past few months [with the PA sessions], my mom became happier, more aware, more articulate and better socialized. I know from past experience that walking has always helped her mood and cognition. (SDM, interview R3)

### 3.3 The perceived barriers to implementing **person‐centred care** in PA for residents with dementia

SDM and LTC home staff reported three main barriers to providing person‐centred care to residents with dementia: (1) a lack of time, (2) perceived resident aggression towards LTC home staff and (3) inadequate PA offerings in the nursing homes. These barriers to person‐centred care also permeated through all aspects of resident care. The lack of time prevented staff from meeting the needs of residents:

He [the resident] can express his needs and wants and his personality but due to time constraints—we want to spend that time with him—but we can't spend the time that we would like to talk to him. (LTC home staff, interviewee R11)

The time constraints were evident to SDMs as well. Although SDMs acknowledged the workload of staff, they viewed the lack of time and attention to their residents as an aggravating factor to responsive behaviours. SDMs expect that LTC home staff should ask residents on a humanistic level if they were ‘okay’ and to consider the pace and speed of the resident and then adjust accordingly during interactions:

The people here don't take any time out of their day to do anything for him or to make sure he's OK. They do what they have to and then go. He [the resident] doesn't respond well to being rushed. (SDM, interviewee R5)

The second barrier was staff's experience with responsive behaviours such as aggression from residents during the provision of PA (i.e., walking the resident down the hall). As a form of responsive behaviour, residents may verbally or physically abuse the LTC home staff and threaten to harm themselves during the PA, which is a challenge for staff:

[Resident] …she's unpredictable so it is hard to work with her if you don't know her. This can be hard for some staff who are not used to her. She gets very upset; she'll scream at you and say things that are not true; so it is important to know how to work with her and to give her what she needs so it won't be so hard for her or the staff. (LTC home staff, interviewee R14)

She is a very difficult resident to care for; she has many responsive behaviors and is very violent. She screams all day long and it can be…just too much. (LTC home staff, interviewee R20)

The third barrier was inadequate PA offerings resulting in fewer occasions for residents to experience social, physical or cognitive engagement and stimulation. The majority of SDMs voiced concern about the limited participation in the number and types of activities organized by the nursing homes. Person‐centred care throughout PA cannot be practiced by LTC home staff if opportunities for PA are not plentiful and diverse. SDMs noticed that activities offered in the nursing homes were not catered to the specific residents. One SDM recalled:

With tailored PA, he will be more active, and it will keep him going. He doesn't enjoy the group activities here. They are generalized and are not about things he likes. (SDM, interviewee R5)

## 4 DISCUSSION

To our knowledge, this is the first study to explore SDMs' and LTC home staff's perception of person‐centred care in terms of how it is conceptualized and applied in PA for LTC home residents with dementia. These two important stakeholder groups highlighted two care processes that best espouse person‐centred care—having a sympathetic presence (e.g., seeing and treating the resident as a unique individual) and engagement (e.g., quality of the relationship between resident and caregiver) processes in the McCormack and McCance framework as most reflective of their conceptualization of person‐centred care in PA. The participants consistently described the benefits and importance of person‐centred care in PA for older adults with dementia, with no descriptions of negative changes or resident impacts. Furthermore, stakeholders reported that when these person‐centred behaviours were implemented during PA, they perceived noticeable improvements in the residents' physical, behavioural, cognitive and psychosocial health and the ability to independently perform activities of daily living. While three care processes were not evident through the interviews (e.g., sharing decision‐making; providing holistic care; and, working with the patient's beliefs and values), this does not preclude their value when delivering person‐centred care. Rather, our results suggest that SDMs and PSWs place the greatest emphasis on having a sympathetic presence and engagement. The identified barriers of enacting person‐centred care during PA were the lack of time due to workload, the time‐intensive act of mitigating resident aggression towards staff and a lack of participation in group activities that were offered in LTC homes.

Our results provide actionable items related to person‐centred care for LTC home staff who are uncertain about how to implement this type of care when providing PA. This contribution is helpful to counter the sentiment that person‐centred care is a nebulous ‘catch‐all’ term for anything to do with high‐quality healthcare.^57^, ^58^ A poor understanding of how to operationalize person‐centred care leads to missed opportunities for staff to humanize care^58^—especially during times of PA and movement. As noted by the SDMs, when staff behaviours that reflected person‐centred care were enacted, SDMs noticed positive changes among residents including an increase in the frequency and duration of PA throughout the day, increased function, communication and improved mood. Physical engagement and activity are associated with positive cognitive and physical effects for older adults with dementia.^20^ This can be corroborated by McCormack and McCance's framework that indicates person‐centered processes will lead to positive outcomes such as feelings of well‐being and satisfaction;^40^ a person‐centred care approach facilitates trusting and meaningful relationships that encourage residents to engage more frequently in PA, leading to improved functional mobility.^24^

Beyond the physiological benefits achieved through increased regular and/or spontaneous PA, person‐centred care behaviours enacted during the PA facilitated the development of meaningful social relationships between the residents and the Multifaceted Walking Intervention interventionist (C. H. C.). In turn, this better supports person‐centred culture, which is the ‘formation and fostering of healthful relationships between all care providers, service users and others significant to them in their lives’.^40^ Evidence shows that espousing person‐centred care with residents who have dementia promotes meaningful social relationships, which in turn reduces depression and agitation in residents.^4^ Participants reported that they felt that the person‐centred care approach was embraced by staff when they were able to demonstrate actions that engaged with residents' personhood, such as talking about past events in the resident's life and providing choices related to PA, and showing genuine compassion and care,^24^ and these actions were categorized as part of the sympathetic process as well as engagement. This is consistent with previous work that suggests that the incorporation of personal preferences into care leads to feelings of support and appreciation,^59^ forming the foundation of social relationships. SDMs and LTC home staff in this study both observed a positive effect of residents during and after the PA sessions in comparison to routine care that does not include individualized PA sessions. Based on their intimate knowledge about the residents, SDMs felt that PA positively contributed to the residents' meaningful social relationships to increase their social interactions, reducing loneliness,^33^ frustration and stress,^4^ and improved well‐being in residents with dementia.^33^ Three care processes from the McCormack and McCance framework were not highlighted by study participants; however, this does not imply that they were not important. It could be that these other care processes were already included in the standard of care, such as family involvement in shared decision‐making,^60^ and the two highlighted processes require more emphasis.

With respect to barriers, SDMs recalled numerous occasions where they witnessed residents being rushed and the LTC home unable to take to engage in person‐centred care due to time and low staffing constraints. These perceptions and responses about the barriers to person‐centred care are not unique to our study participants,^61^ and such time constraints have only been amplified with the COVID‐19 pandemic and the increased turnover of LTC home staff.^62^, ^63^ Moreover, staff in our study also reported resident aggression towards them as a significant challenge to providing person‐centred care.^64^ Staff who experienced aggressive behaviours from residents are more likely to report feeling emotional exhaustion and negative feelings with inadequate care, anxiety, depression and absenteeism.^65^, ^66^ This finding provides additional direction to reform working and living conditions for staff and residents in LTC homes, respectively.^67^ Most LTC homes work within the biomedical model and primarily focus on staff completing ‘tasks’ rather than prioritizing actions that are aligned with person‐centred care and outcome achievement.^67^ Newer innovative models of care (e.g., Eden Alternative, GreenHouse Model, Butterfly Model) that are based on developing strong resident–caregiver relationships and mutual respect contribute to an environment that is receptive to residents' needs.^68^ A supportive organizational culture and work environment allows staff to feel more satisfied with care^68^ and decreases resident aggression, which in turn can lead to improved residents and staff outcomes.

The methodological strengths of this study included the fact that the participants were recruited from multiple LTC homes that differed in size and resources. Home 1 is a large 350‐bed facility, and Home 2 is a mid‐sized 128‐bed LTC home with a foundation that provided additional financial resources earmarked to improve resident socialization, for example, Home 2 is able to support more social events than Home 1. The inclusion of both SDM and staff participants enriches our understanding of person‐centred care by combining these two adjacent caregiver perspectives. We had a 100% participation rate from recruited SDMs and staff participants; they were very motivated to discuss the importance of person‐centred care. Additionally, our SDM sample was consistent with the national profile of informal caregivers in Canada,^69^ Australia,^70^ across the European union^71^ and Asia,^72^ where the large majority are female and older than 45 years of age. The McCormack and McCance framework appropriately guided coding to delineate environmental barriers, the key care processes and perceived benefits (outcomes) related to person‐centred care. The qualitative evidence from SDMs and LTC home staff regarding person‐centred care delivery challenges provides rich contextualization and should be further investigated to understand its impact on residents. One study limitation may be that there was a lack of ethnic diversity among SDMs, which may have introduced underlying cultural assumptions about how PA is conceptualized and the definition of aggression. Further, we were unable to include additional methods such as observations or interviews with dyads of SDMs and staff because it was not logistically feasible, given the scheduling of LTC home staff and SDMs. Lastly, this study does not include the preferences and perspectives of the residents themselves and is an area for future research.

### 4.1 Implications for practice

By identifying the specific behaviours and actions that align best with SDM and staff expectations about person‐centred care, our results have the potential to increase this type of care during PA via the development of education, training and more effective public strategies to support staffing levels and capacity. Findings suggest that both SDMs and staff appreciate being able to connect and engage with residents on a personal level. It is notable for staff to hold this sentiment despite the heavy workloads that they face. Given the challenging context of low staffing, this study suggests that any opportunities that enable meaningful personal connection is valued by residents, SDMs and staff. Providing SDMs and staff with the opportunity to collaborate on the development of PA programmes for residents could help focus care on resident needs, and improve delivery of person‐centred care.

In terms of public policy, there is an urgent need to address the underlying causes like poor work organization, heavy workloads and staff‐to‐resident ratios, which in turn will reduce the identified barriers to person‐centred care (lack of time, resident aggression, inadequate PA offerings). Inadequate staff time to provide relational care can increase residents' aggression towards staff.^64^, ^73^ Ninety percent of LTC home workers report experiencing violence from residents^74^ and few opportunities for person‐centred relational care. Additional aspects of the LTC environment can impact the ability to support person‐centred care such as the technological infrastructure and resources in the home.^75^ LTC homes themselves need to be held more accountable for ensuring a safe work environment and to improve work conditions that can foster person‐centred care.

## 5 CONCLUSION

This study has provided an in‐depth exploration about how SDMs and LTC home staff perceive person‐centred care during PA with residents with dementia. The results provide insights into the actions that most reflect person‐centred care to SDMs and LTC home staff—sympathetic processes and engagement—in addition to the barriers and benefits related to person‐centred care. Operationalization and identification of the actions related to person‐centred care provide a greater understanding about the provision of these actions during PA to optimize residents' physical health and well‐being.

## CONFLICT OF INTERESTS

The authors declare that there are no conflict of interests.

## Supporting information

Supporting information.

Supporting information.

## Data Availability

The data that support the findings of this study are available from the corresponding author upon reasonable request.
