# Supplementary material for: Perspectives of substitute decision‐makers and staff about person‐centred physical activity in long‐term care
Source: Health Expect. 2021 Nov 8;25(5):2155–65. doi: 10.1111/hex.13381 (PMC9615080; doi:10.1111/hex.13381)
Supplement: Supplementary file 1 — Supporting information. [file HEX-25--s001.docx]

# Appendix A

# Semi-Structured Interview Guide

*Note: As this is semi-structured interview, the wording and order of questions might be slightly changed based on the flow of interview*.

# Section A: Introduction

We would like to get a better understanding of the care processes that you think best exemplify person-centered care when delivering physical activity (PA) to LTC residents with dementia.

Throughout this interview, remember that there are no right or wrong answers. If there is a word that is unfamiliar to you or if a question is unclear, please ask me about it. You may stop the interview at any time. If there is a question that you do not feel comfortable with answering, you do not need to answer it. Just let me know. Now I would like to ask you a few questions about yourself and technology.

We would like to record the interview to ensure the accuracy of our data. Do we have your permission?

- Your name will be removed from the transcript
- Only the research team on this project will have access to the audio recording and transcript of this interview.

*[If affirmative, start recording]*

1. Do you agree to participate in this interview?
   - Please note that you can stop at any time.
   - You do not have to answer all our questions

*[If participant declines to be audio-recorded]*

1. OK, I understand. I will still ask you all the same questions and will write down your answers instead. Is this alright with you?

# Section B: Questions for Substitute Decision Makers (SDMs)

1. Please describe how important is “person-centered care” (PCC) as it relates to the care of your loved one and other nursing home residents with dementia?
2. Do you think combining person centered care and physical activity (e.g. walking) will influence the mobility of your loved one with dementia? Why? (Please describe any impact you think it may or may not have)
3. Do you think combining person centered care and physical activity (e.g. walking) will influence your loved one's ability to perform daily care activities (e.g. toileting, transferring) if they have dementia? Why?
4. Describe your values, beliefs, or judgments about the effects of combining PCC and physical activity on the quality of life in residents who have dementia?
5. With respect to the quality of life in residents who have dementia, what are barriers to providing person-centered care to residents with dementia?

# Section C: Questions for Staff

1. What is it like to care for this resident with dementia?
2. Please describe how important is “person-centered care” as it relates to the care of this resident and other nursing home residents with dementia?
3. Do you think combining person centered care and physical activity (e.g. walking) will influence the mobility of residents who have dementia? Why?
4. Do you think combining person centered care and physical activity (e.g. walking) will influence a resident’s ability to perform daily care activities (e.g. toileting, transferring) if they have dementia? Why?
5. Describe your values, beliefs, or judgments about the effects of regular walking and talking on the quality of life in residents who have dementia?
6. What are barriers to providing person-centered care to residents with dementia?

# Section D: Conclusion

*We will conclude each interview session by doing the following:*

- *Ask if there is anything else the participant wants to share about how they believe person-centered care is best delivered*
- *Ask the participants if they have any feedback for improving the interview.*
- *Ask if we could contact them again to clarify the content of the interview if needed.*

If a participant would like to withdraw:

- If a participant would like to withdraw and does not want us to use the collected data, they can do so at any moment.
