# Supplementary material for: Perspectives of substitute decision‐makers and staff about person‐centred physical activity in long‐term care
Source: Health Expect. 2021 Nov 8;25(5):2155–65. doi: 10.1111/hex.13381 (PMC9615080; doi:10.1111/hex.13381)
Supplement: Supplementary file 2 — Supporting information. [file HEX-25--s002.docx]

# Appendix B

ID: ___________________ Date: __________________

**Demographic information of the Substitute Decision Maker (SDM)**

This information will be kept confidential. Data will be analyzed and reported as a group. Your name and personal information will never be used. Please fill out the following information:

Gender (please circle): M F

Your relationship to the resident: ______________________

Your year of birth: ______________

Your highest level of education: ______________

On average, how many times do you visit the resident a week?

1 day a week 2 days a week 3 days a week 4 days a week

5 days a week 6 days a week 7 days a week

Please rate on a scale of 1 to 10 how well you know the resident:

1 (LEAST) 2 3 4 5 6 7 8 9 10 (MOST)

What is the **resident’s** highest level of education? ________________

Do you employ a **private caregiver** to provide care for your loved one? N or Y

If yes, please indicate how often the caregiver comes to visit your relative: ________________________

Would you like to receive the *overall* results of the study? Yes or No

Would you like to receive the results *specific to your family member*? Yes or No

Results will be emailed. Please provide your email address: _____________________________
